# Hysteria—Dog

**Published:** 1897-05

**Authors:** H. W. Smith

**Affiliations:** Mississippi


### HYSTERIA—DOG.

By H. W. Smith, V.S.,
MISSISSIPPI.

I was called to see a dog said to be mad. It was a fox-terrier, and said to be of high pedigree. It was noticed at play chasing birds, etc. After a time it seemed to be taken with a fit of barking. The dog was caught and put in a stable. A druggist and several others were called, who pronounced the dog mad. On my arrival I noticed that the dog did not appear to be in any way vicious, but was acting in a manner that any well-pleased dog might be expected to do—running around the stable, jumping up the wall, barking and wagging its tail all the time. I was told that he had acted in that fashion for three hours, during which time he had not stopped barking once.

I stated that in my opinion the dog was simply overexcited, not mad—in fact, that it had hysteria, and prescribed chloral hydrate, grs. x. The effect was almost immediate calmness. I left a dose of ten grains, to be given in half an hour.

